# Spinal fusion surgery use among adults with low back pain enrolled in a digital musculoskeletal program: an observational study

**DOI:** 10.1186/s12891-024-07573-0

**Published:** 2024-07-05

**Authors:** Sandhya Yadav, Laura S. Gold, Qasim Hassan Zaidi, Raymond Hwang, Louie Lu, Grace Wang

**Affiliations:** 1 Clinical Research, Hinge Health, Inc., 455 Market Street, San Francisco, CA 94105 USA; 2 Clinical Learning, Evidence and Research Center, University of Washington, Seattle, WA USA; 3 Signature Medical Group, St. Louis, MO USA; 4 Weitzman Institute, Middletown, CT USA

**Keywords:** Digital MSK, Spinal fusion, Medical claims, Lumbar fusion, Low back pain

## Abstract

**Objectives:**

To compare 12-month spinal fusion surgery rates in the setting of low back pain among digital musculoskeletal (MSK) program participants versus a comparison cohort who only received usual care.

**Study Design:**

Retrospective cohort study with propensity score matched comparison cohort using commercial medical claims data representing over 100 million commercially insured lives.

**Methods:**

All study subjects experienced low back pain between January 2020 and December 2021. Digital MSK participants enrolled in the digital MSK low back program between January 2020 and December 2021. Non-participants had low back pain related physical therapy (PT) between January 2020 and December 2021. Digital MSK participants were matched to non-participants with similar demographics, comorbidities and baseline MSK-related medical care use. Spinal fusion surgery rates at 12 months post participation were compared.

**Results:**

Compared to non-participants, digital MSK participants had lower rates of spinal fusion surgery in the post-period (0.7% versus 1.6%; *p* < 0.001). Additionally, in the augmented inverse probability weighting (AIPW) model, digital MSK participants were found to have decreased odds of undergoing spinal fusion surgery (adjusted odds ratio: 0.64, 95% CI: 0.51–0.81).

**Conclusions:**

This study provides evidence that participation in a digital MSK program is associated with a lower rate of spinal fusion surgery.

**Supplementary Information:**

The online version contains supplementary material available at 10.1186/s12891-024-07573-0.

## Key messages

• There is limited research comparing spinal fusion surgery rates in digital musculoskeletal (MSK) program participants versus those receiving usual care for low back pain.

• This study shows that participation in a digital MSK program was associated with 56% reduction in spinal fusion surgery rates.

• These findings highlight the potential of digital MSK programs to reduce spinal fusion surgeries, offering a new direction for reshaping clinical practice in low back pain management.

**Supplementary Information:**

The online version contains supplementary material available at 10.1186/s12891-024-07573-0.

## Background

Musculoskeletal (MSK) conditions are a leading cause of disability and cost in the United States (US) [1]. The rates of low back pain, and other MSK disorders in the US are among the highest in the world [1]. Rates of spine surgery worldwide have trended upward, with the highest incidence in the US, raising concerns about potential overuse [2, 3]. The number of elective lumbar fusions for low back pain increased 276% in the US from 2002 to 2014, resulting in economic burden on patients, payers and society [4, 5]. Aggregate hospital costs for elective lumbar fusion increased 177% during these 12 years, exceeding $10 billion in 2015 [4]. Average hospital charges per stay for a spinal fusion in 2020 were $164,543 in the US [6].

While low back pain can significantly impact quality of life, a number of treatment options are available. Physical therapy (PT) has been demonstrated to be an effective first line treatment for patients with low back pain [7]. PT programs can reduce low back pain and maximize function by improving flexibility, muscular strength, and endurance, and have been associated with a lower likelihood of undergoing spinal fusion surgery within one year [8]. The World Federation of Neurosurgical Societies (WFNS) Spine Committee analyzed treatment options for patients with degenerative disease of the spine without neurologic symptoms and recommended a conservative approach based on therapeutic exercise as the first line of treatment [9].

Increasingly, conservative care approaches are delivered digitally. Evidence suggests that digital MSK care programs (hereafter, digital MSK program) are as effective in improving MSK-related pain, function, and surgery intention outcomes as traditional in-person care [10–15]. Whether a digital MSK program is associated with a lower incidence of spinal fusion surgery among individuals with low back pain remains unknown.

### Objective

To address this evidence gap, this study’s primary objective was to examine whether digital MSK program participants with low back pain had lower rates of spinal fusion surgery compared to non-participants receiving usual care for low back pain in a commercially insured population.

## Methods

### Study design and data source

This was an observational study using HIPAA-compliant, de-identified medical claims data sourced from a claims database that comprised over 100 million commercially insured members across all US states and territories. We compared spinal fusion surgery rates over 12 months among digital MSK program participants with low back pain to a propensity score-matched non-participant cohort (herein, nonparticipants) who received usual care.

### Study subjects

All subjects experienced low back pain between January 2020 and December 2021. Digital MSK participants were engaged in the digital MSK back program between January 2020 and December 2021. Nonparticipants had at least one low back pain (ICD-10-CM diagnosis code M54.5) related PT visit between January 2020 and December 2021 (hereafter, index event).

Only subjects aged 40–64 years were included due to the higher prevalence of spinal fusions in this age group [16]. Inclusion criteria required continuous enrollment in a health plan for at least 12 months both before and after commencing the digital MSK program or experiencing their index event. All subjects had at least one back pain-related MSK service in the 12 months before starting the digital MSK program or index event (hereafter, 12-month baseline period).

Study subjects with a spinal fusion surgery in the 12-month baseline period were excluded. We excluded subjects who had an orthopedic surgeon or neurosurgeon visit in the 3 months before starting the digital MSK program or the index event. We also excluded subjects with cancer, pregnancy, or childbirth, or outlier total annual medical cost (>$500,000) during the study period.

### Exposure: digital MSK program

**Fig. 1:**
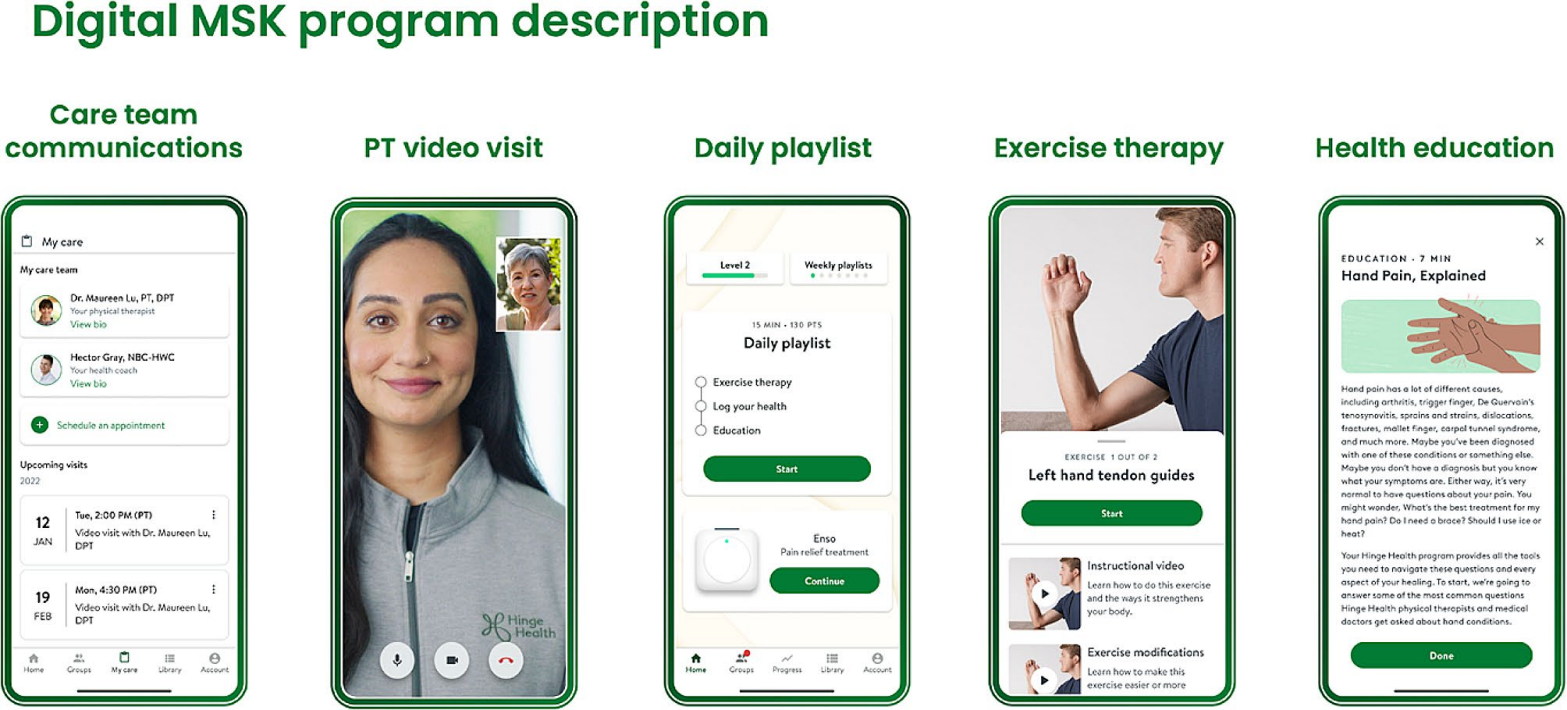
Digital MSK program description

The digital MSK program is a product of Hinge Health and is described elsewhere [17]. Briefly, this program was a health benefit for employees and dependents offered through their employers. All individuals aged 18 and over were eligible, and participation was voluntary. The program’s goal was to help participants manage back pain by offering exercise therapy, education, and virtual access to a care team including physical therapists and personal health coaches (Fig. 1). It provided members with tablet computers with a program app that used “playlists” to present three to eight different stretching, strengthening, balance and mobility exercises via animations and videos. It also provided wearable motion sensors (InvenSense MPU-6050, TDK Electronics, Tokyo, Japan) that gave feedback through the app about range of movement and repetitions. After exercises, members received educational resources and support from certified health coaches.

### Primary outcome: spinal fusion surgery

The primary outcome of this study was whether a subject had a spinal fusion surgery (excluding revision surgery) in the 12 months after starting the digital MSK program or having their index event. We used the Current Procedural Terminology (CPT)/Healthcare Common Procedure Codes (HCPCS), Diagnosis related group (DRG), and International Classification of Diseases, 10th Revision, Procedure Coding System (ICD-10-PCS) codes in the medical claims to identify spinal fusion and revision surgeries (online appendix).

### Covariates

We included subjects’ demographic characteristics, comorbidity burden and healthcare service utilization in the 12 months before starting the digital MSK program or having their index event as covariates.

Demographic characteristics included: age group (40–49, 50–64 years at the time of data extraction), sex (male, female), census division (New England, Middle Atlantic, East North Central, West North Central, South Atlantic, East South Central, West South Central, Mountain, Pacific) [17], rurality (rural, urban).

Comorbidity burden included the weighted Elixhauser score, degenerative spinal diagnosis, and concurrent MSK conditions. We used Elixhauser coding algorithms available for International Classification of Diseases, Tenth Revision, Clinical Modification (ICD-10-CM) codes [18]. Weighted Elixhauser scores were computed using primary ICD-10-CM diagnosis in medical claims from January 2019 and December 2022 and further stratified into groups (< 0, 0, 1–4, ≥ 5) [19]. In addition to low back pain, we identified concurrent chronic MSK conditions in the knee, shoulder, hip and neck from medical claims in the three months before program start month or index event month (see online appendix for codes used to define each concurrent MSK condition). Degenerative spinal diagnosis during the 12-month baseline period was determined using ICD-10-CM diagnosis codes (online appendix).

To identify and categorize MSK-related health care use during the baseline period, we used Restructured BETOS Classification System (RBCS) [20]. We included the following variables: number of back pain related injections at baseline (0,1–5, 6+); number of back pain related provider visits at baseline (0,1–5, 6+); number of back pain related imaging at baseline (0,1–5, 6+); number of back pain related PT visits at baseline (0,1–5, 6+); and recent MSK care (0,1) defined as back pain related injection, imaging, provider visit or PT visit in the 3 months before index event or starting the digital MSK program.

### Statistical methods

To address confounding variables, we matched digital MSK participants to similar non-participants using propensity scores. First, we calculated a propensity score for each subject using a logit model with the following covariates: demographics (i.e., age group, sex, census division, rurality), weighted Elixhauser score and degenerative spinal diagnosis at baseline, concurrent MSK conditions (i.e. knee, shoulder, hip and neck) in the three months before the index month, baseline MSK-related health care use (variables described above). Next, we matched non-participants to digital MSK participants based on calculated propensity scores, using 1:1 nearest neighbor matching without replacement. Covariate balance after matching was assessed using standardized mean differences (appendix, Figure S1). The final analytic sample included 3424 matched pairs.

To describe study subjects, we generated descriptive statistics for the matched sample for baseline factors. We applied chi-square tests for categorical variables and t-tests for continuous variables to evaluate differences between cohorts.

As a robustness check, we employed Augmented Inverse Propensity Weighting (AIPW) in addition to the main analysis. AIPW is an extension of inverse probability weighting (IPW) that can help reduce selection bias in observational studies by adjusting for confounding variables and accounting for unmeasured confounding [21]. We also conducted an additional sensitivity analysis using a multivariate logistic regression model including the set of covariates listed above. Statistical significance was tested at two-sided *P* < 0.05. Model fit was assessed using the Hosmer-Lemeshow goodness-of-fit test.

## Results

### Descriptive results

After matching, there were no statistically significant differences on any of the baseline characteristics between the digital MSK cohort and the non-participant cohort (Table 1).

Our matched population had more females than males and more than 65% were aged 50 years or older. Geographically, a significant concentration of subjects resided in the East North Central, South Atlantic, and West South Central regions. Most were urban residents. About 45% had concurrent MSK conditions in the three months prior to the index event. More than 90% had a weighted Elixhauser score equal to 0 or less.

Study subjects used a range of health care services in the baseline period. MSK-related provider visits were the most frequent service among the study subjects with over 70% having provider visits during the baseline period. PT visits were also common, with more than 50% of the subjects having PT visits. A small proportion, about 15%, had injections and 47% had imaging.

**Table 1:** Baseline characteristics of study subjects after matching

Characteristics	Non-participants	Digital MSK participants	*p*-value
	*N* = 3,424	*N* = 3,424	
	*n* (%)	*n* (%)	
**Demographics**			
Age group			
40–49	1144 (33.4%)	1146 (33.5%)	0.98
50–64	2280 (66.6%)	2278 (66.5%)	
Gender			
Male	1614 (47.1%)	1597 (46.6%)	0.698
Female	1810 (52.9%)	1827 (53.4%)	
Census region			
New England	90 (2.6%)	86 (2.5%)	0.972
Middle Atlantic	165 (4.8%)	157 (4.6%)	
East North Central	756 (22.1%)	784 (22.9%)	
West North Central	202 (5.9%)	192 (5.6%)	
South Atlantic	647 (18.9%)	618 (18.0%)	
East South Central	176 (5.1%)	174 (5.1%)	
West South Central	577 (16.9%)	589 (17.2%)	
Mountain	236 (6.9%)	250 (7.3%)	
Pacific	575 (16.8%)	574 (16.8%)	
Rurality			
Rural	377 (11.0%)	386 (11.3%)	0.759
Urban	3047 (89.0%)	3038 (88.7%)	
**Musculoskeletal Comorbidity**			
Concurrent MSK			
No	1872 (54.7%)	1902 (55.5%)	0.481
Yes	1552 (45.3%)	1522 (44.5%)	
Spondylosis diagnosis			
No	2389 (69.8%)	2361 (69.0%)	0.479
Yes	1035 (30.2%)	1063 (31.0%)	
Spondylolysis diagnosis			
No	3419 (99.9%)	3413 (99.7%)	0.211
Yes	5 (0.1%)	11 (0.3%)	
Spondylolisthesis diagnosis			
No	3345 (97.7%)	3329 (97.2%)	0.249
Yes	79 (2.3%)	95 (2.8%)	
Radiculopathy diagnosis			
No	2326 (67.9%)	2321 (67.8%)	0.918
Yes	1098 (32.1%)	1103 (32.2%)	
Stenosis diagnosis			
No	3418 (99.8%)	3418 (99.8%)	1
Yes	6 (0.2%)	6 (0.2%)	
Weighted Elixhauser Comorbidity Score			
<0	882 (25.8%)	870 (25.4%)	0.862
0	2307 (67.4%)	2307 (67.4%)	
1–4	207 (6.0%)	213 (6.2%)	
>=5	28 (0.8%)	34 (1.0%)	
**Baseline MSK utilization**			
Recent MSK service (3 mo baseline)			
No	1813 (52.9%)	1779 (52.0%)	0.425
Yes	1611 (47.1%)	1645 (48.0%)	
Injections (12 mo baseline)			
0	2920 (85.3%)	2876 (84.0%)	0.258
1 to 5	454 (13.3%)	486 (14.2%)	
6 or more	50 (1.5%)	62 (1.8%)	
Imaging (12 mo baseline)			
0	1773 (51.8%)	1801 (52.6%)	0.507
1 to 5	1631 (47.6%)	1597 (46.6%)	
6 or more	20 (0.6%)	26 (0.8%)	
Physical therapy (12 mo baseline)			
0	1704 (49.8%)	1661 (48.5%)	0.58
1 to 5	897 (26.2%)	922 (26.9%)	
6 or more	823 (24.0%)	841 (24.6%)	
Provider visit (12 mo baseline)			
0	917 (26.8%)	933 (27.2%)	0.468
1 to 5	2355 (68.8%)	2320 (67.8%)	
6 or more	152 (4.4%)	171 (5.0%)	

Table note: *p < 0.05. Baseline (3 mo) is defined as the 3 months before starting the digital program or the index event. Baseline (12 mo) is defined as the 12 months before starting the digital program or the index event. Across all the matching covariates, there are no statistically significant differences between the matched cohorts

### Main findings

**Fig. 2:**
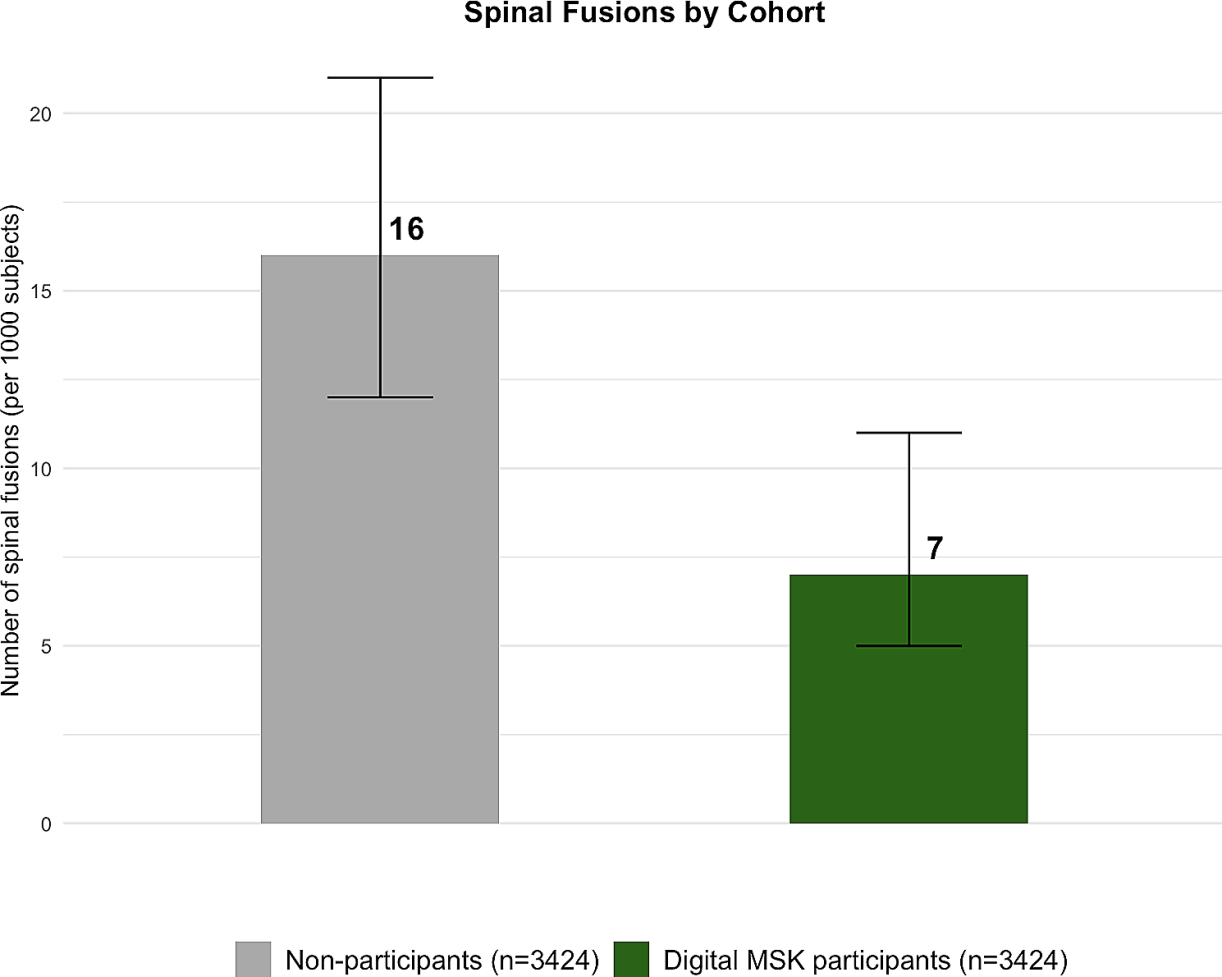
Spinal fusion surgery rates in the post-period, after matching. *Note*: Error bars indicate 95% CI. Estimates are significant at *p* < 0.001

Table 2 presents a comparison of spinal fusion utilization in the 12-month post-period for digital MSK participants and non-participants. In the post-period, only 0.7% of digital MSK participants underwent spinal fusion surgery, while a more substantial 1.6% of the matched non-participants opted for spinal fusion surgery (Fig. 2).

**Table 2:** Spinal fusion surgery rates in the post-period, after matching

	Non-participants	Digital MSK participants	
	(*N* = 3,424)	(*N* = 3,424)	
Spinal fusion in the post-period	*n* (%, 95% CI)	*n* (%, 95% CI)	*p*-value
Yes	54 (1.6, 1.20–2.07)	24 (0.7, 0.46–1.06)	< 0.001
No	3370 (98.4, 97.9–98.8)	3400 (99.3, 98.9–99.5)	

Results from the AIPW model were consistent with the main analysis. Compared to non-participants, digital MSK participants had significantly lower odds of spinal fusion surgery (adjusted odds ratio: 0.64, 95% CI: 0.51–0.81), suggesting that our results were not sensitive to unmeasured confounding, (Table S1, online appendix). These results are also supported by the multivariable regression analysis, which also found lower odds of spinal fusion surgery, both in the unadjusted analysis (OR: 0.44, 95% CI: 0.27–0.71, *p* = 0.001) and after adjusting for demographics, MSK comorbidity profile, and baseline healthcare utilization (adjusted OR: 0.43, 95% CI: 0.26–0.69, *p* = 0.001), (Table S2, online appendix).

## Discussion

This study used a large commercial claims dataset to examine spinal fusion surgery rates among individuals who participated in a digital MSK program for low back pain compared to non-participants who received usual care. This analysis showed lower spinal fusion rates among digital MSK participants, specifically, 56% fewer digital MSK participants underwent spinal fusion surgery than non-participants at 12 months. These findings add evidence that digital MSK program is associated with lower spinal fusion surgery rates for individuals experiencing low back pain. This aligns with prior studies that have highlighted the efficacy of exercise as treatment for low back pain, thereby providing a plausible explanation for the lower odds of surgical intervention observed in this study.

These results are consistent with recent systematic reviews that demonstrated statistically significant improvements in clinical outcomes when comparing digital MSK programs to usual care or active controls (e.g., health education) [12, 13, 22–24]. In addition, we have previously reportedly on the significant improvements in self-reported pain and function among participants in a digital MSK program [25–28]. We theorized that as patients experience tangible improvements in pain and functional capacity through such programs, their inclination to explore surgical options diminish. Reasons for this may include the holistic nature of the program, which included physical therapists and health coaches to address the physical and behavioral components of pain, and also fostered patient confidence in the non-surgical management.

When interpreting the results of this study, its strengths and limitations should be considered. To our knowledge, this is the first study comparing a digital MSK program for spinal fusion surgery incidence against a nonparticipant cohort using a universal sample of members with low back pain from a commercial database that included over 100 million lives. The strengths include a large sample size, and findings are generalizable to adults with low back pain and employer-based medical coverage. One limitation of our study is that the retrospective observational nature does not demonstrate causal effect of the digital MSK program on spinal fusion surgery. The analysis is subject to potential selection bias such as patient preference for conservative care versus surgery, patients with worse pain and lower function may be less likely to engage with digital health platforms than usual care.

Second, some key confounding variables were not included in the analysis. Several factors, such as race/ethnicity and functional status, merit consideration. Specific race/ethnic groups may experience a disproportionate impact of MSK pain. Earlier research revealed that expected pain and functional improvement were associated with decisions to have MSK surgery [29–31].

Third, in order to maintain claims data de-identification, we were not able to link member engagement data (e.g. counts of exercise) with the medical claims data. These estimates would be interpreted as in an “intent-to-treat” analysis, although it would be helpful to test whether higher engagement was associated with lower surgery use. Additionally, we were unable to determine whether the digital MSK program completely prevented or simply delayed surgeries beyond 12 months of follow-up.

To address these limitations, future research could include prospectively designed RCTs addressing causation to further delineate the efficacy of a digital MSK program on decreasing spinal fusion surgery rates and associated cost impact. While this study examined spinal fusion surgery rates at 12 months, longer follow-up would increase our understanding of the sustained effects of digital MSK programs on reducing the need for surgical interventions.

## Conclusions

In conclusion, this study provides evidence supporting the role of digital MSK programs in managing low back pain and its association with lower incidence of spinal fusion surgeries. Further research will enhance our understanding of digital health interventions in the management of MSK conditions with the ultimate goal of improving healthcare delivery and patient outcomes.

### Electronic supplementary material

Below is the link to the electronic supplementary material.

Supplementary Material 1

## Data Availability

The datasets used and/or analyzed during the current study are available from the corresponding author on reasonable request.

## Abbreviations

MSK: Musculoskeletal
PT: Physical Therapy
AIPW: Augmented inverse probability weighting
US: United States
WFNS: World Federation of Neurosurgical Societies
CPT: Current Procedural Terminology
HCPCS: Healthcare Common Procedure Codes
DRG: Diagnosis related group
PCS: Procedure Coding System
RBCS: Restructured BETOS Classification System

## References

[1] Vos T, Lim SS, Abbafati C, Abbas KM, Abbasi M, Abbasifard M (2020). Global burden of 369 diseases and injuries in 204 countries and territories, 1990–2019: a systematic analysis for the global burden of Disease Study 2019. Lancet.

[2] Reisener MJ, Pumberger M, Shue J, Girardi FP, Hughes AP (2020). Trends in lumbar spinal fusion—a literature review. J Spine Surg.

[3] Kim P, Kurokawa R, Itoki K (2010). Technical advancements and utilization of spine surgery. Neurol Med Chir (Tokyo).

[4] Martin BI, Mirza SK, Spina N, Spiker WR, Lawrence B, Brodke DS (2019). Trends in lumbar Fusion Procedure Rates and Associated Hospital costs for degenerative spinal diseases in the United States, 2004 to 2015. Spine.

[5] Deng H, Yue JK, Ordaz A, Suen CG, Sing DC (2021). Elective lumbar fusion in the United States: national trends in inpatient complications and cost from 2002–2014. J Neurosurg Sci.

[6] HCUPnet Data Tools. – Healthcare Cost and Utilization Project (HCUPnet). [cited 2023 Aug 10]. https://datatools.ahrq.gov/hcupnet/

[7] Degenerative Disc Disease. SERC. [cited 2023 Jan 24]. https://serc.urpt.com/degenerative-disc-disease/

[8] Fritz JM, Lurie JD, Zhao W, Whitman JM, Delitto A, Brennan GP (2014). Associations between physical therapy and long-term outcomes for individuals with lumbar spinal stenosis in the SPORT study. Spine J.

[9] Fornari M, Robertson SC, Pereira P, Zileli M, Anania CD, Ferreira A (2020). Conservative Treatment and Percutaneous Pain Relief techniques in patients with lumbar spinal stenosis: WFNS Spine Committee Recommendations. World Neurosurg X.

[10] Valentijn PP, Tymchenko L, Jacobson T, Kromann J, Biermann CW, AlMoslemany MA (2022). Digital Health Interventions for Musculoskeletal Pain conditions: systematic review and Meta-analysis of Randomized controlled trials. J Med Internet Res.

[11] Latif-Zade T, Tucci B, Verbovetskaya D, Bialkin E, Ng B, Heddon S (2021). Systematic review shows Tele-Rehabilitation might achieve comparable results to Office-Based Rehabilitation for decreasing Pain in patients with knee osteoarthritis. Med Kaunas Lith.

[12] Seron P, Oliveros MJ, Gutierrez-Arias R, Fuentes-Aspe R, Torres-Castro RC, Merino-Osorio C (2021). Effectiveness of Telerehabilitation in Physical Therapy: a Rapid Overview. Phys Ther.

[13] Xie SH, Wang Q, Wang LQ, Wang L, Song KP, He CQ (2021). Effect of Internet-Based Rehabilitation Programs on Improvement of Pain and physical function in patients with knee osteoarthritis: systematic review and Meta-analysis of Randomized controlled trials. J Med Internet Res.

[14] Chen T, Or CK, Chen J (2021). Effects of technology-supported exercise programs on the knee pain, physical function, and quality of life of individuals with knee osteoarthritis and/or chronic knee pain: a systematic review and meta-analysis of randomized controlled trials. J Am Med Inf Assoc JAMIA.

[15] Safari R, Jackson J, Sheffield D (2020). Digital Self-Management interventions for people with osteoarthritis: systematic review with Meta-analysis. J Med Internet Res.

[16] Verla T, Adogwa O, Toche U, Farber SH, Petraglia F, Murphy KR (2016). Impact of increasing age on outcomes of spinal Fusion in Adult Idiopathic Scoliosis. World Neurosurg.

[17] Wang G, Lu L, Gold LS, Bailey JF (2023). Opioid initiation within one year after starting a Digital Musculoskeletal (MSK) program: an Observational, Longitudinal Study with Comparison Group. J Pain Res.

[18] Gasparini A (2018). Comorbidity: an R package for computing comorbidity scores. J Open Source Softw.

[19] van Walraven C, Austin PC, Jennings A, Quan H, Forster AJ (2009). A modification of the Elixhauser Comorbidity measures into a Point System for Hospital Death using Administrative Data. Med Care.

[20] Restructured BETOSC. System - Centers for Medicare & Medicaid Services Data. [cited 2023 Aug 9]. https://data.cms.gov/provider-summary-by-type-of-service/provider-service-classifications/restructured-betos-classification-system

[21] Kurz CF (2022). Augmented inverse probability weighting and the double robustness property. Med Decis Mak.

[22] Du S, Liu W, Cai S, Hu Y, Dong J (2020). The efficacy of e-health in the self-management of chronic low back pain: a meta analysis. Int J Nurs Stud.

[23] Suso-Martí L, La Touche R, Herranz-Gómez A, Angulo-Díaz-Parreño S, Paris-Alemany A, Cuenca-Martínez F. Effectiveness of Telerehabilitation in Physical Therapist Practice: an Umbrella and Mapping Review with Meta–Meta-Analysis. Phys Ther. 2021;pzab075. 10.1093/ptj/pzab075 PMC7928612 33611598

[24] Gava V, Ribeiro LP, Barreto RPG, Camargo PR. Effectiveness of physical therapy given by telerehabilitation on pain and disability of individuals with shoulder pain: a systematic review. Clin Rehabil. 2022;02692155221083496. 10.1177/02692155221083496 35230167

[25] Wang G, Yang M, Hong M, Krauss J, Bailey JF (2022). Clinical outcomes one year after a digital musculoskeletal (MSK) program: an observational, longitudinal study with nonparticipant comparison group. BMC Musculoskelet Disord.

[26] Mecklenburg G, Smittenaar P, Erhart-Hledik JC, Perez DA, Hunter S (2018). Effects of a 12-Week Digital Care Program for chronic knee Pain on Pain, mobility, and surgery risk: Randomized Controlled Trial. J Med Internet Res.

[27] Shebib R, Bailey JF, Smittenaar P, Perez DA, Mecklenburg G, Hunter S (2019). Randomized controlled trial of a 12-week digital care program in improving low back pain. Npj Digit Med.

[28] Bailey JF, Agarwal V, Zheng P, Smuck M, Fredericson M, Kennedy DJ (2020). Digital Care for Chronic Musculoskeletal Pain: 10,000 participant longitudinal cohort study. J Med Internet Res.

[29] Liu Q, Chu H, LaValley MP, Hunter DJ, Zhang H, Tao L (2022). Prediction models for the risk of total knee replacement: development and validation using data from multicentre cohort studies. Lancet Rheumatol.

[30] Patel M, Johnson AJ, Booker SQ, Bartley EJ, Palit S, Powell-Roach K (2022). Applying the NIA Health Disparities Research Framework to identify needs and opportunities in Chronic Musculoskeletal Pain Research. J Pain.

[31] Salimy MS, Humphrey TJ, Katakam A, Melnic CM, Heng M, Bedair HS (2023). Which factors are considered by patients when considering total joint arthroplasty? A discrete-choice experiment. Clin Orthop.

