# Supplementary material for: Spinal fusion surgery use among adults with low back pain enrolled in a digital musculoskeletal program: an observational study
Source: BMC Musculoskelet Disord. 2024 Jul 5;25:520. doi: 10.1186/s12891-024-07573-0 (PMC11225358; doi:10.1186/s12891-024-07573-0)

# Appendix

### CPT, DRG, and ICD10 PCS codes that define Spinal fusions (excluding revisions)

### ICD10 PCS codes used to define spinal fusion

0RGA070, 0RGA071, 0RGA07J, 0RGA0A0, 0RGA0A1, 0RGA0AJ, 0RGA0J0, 0RGA0J1, 0RGA0JJ, 0RGA0K0, 0RGA0K1, 0RGA0KJ, 0RGA0Z0, 0RGA0Z1, 0RGA0ZJ, 0RGA370, 0RGA371, 0RGA37J, 0RGA3A0, 0RGA3A1, 0RGA3AJ, 0RGA3J0, 0RGA3J1, 0RGA3JJ, 0RGA3K0, 0RGA3K1, 0RGA3KJ, 0RGA3Z0, 0RGA3Z1, 0RGA3ZJ, 0RGA470, 0RGA471, 0RGA47J, 0RGA4A0, 0RGA4A1, 0RGA4AJ, 0RGA4J0, 0RGA4J1, 0RGA4JJ, 0RGA4K0, 0RGA4K1, 0RGA4KJ, 0RGA4Z0, 0RGA4Z1, 0RGA4ZJ, 0SG0070, 0SG0071, 0SG007J, 0SG00A0, 0SG00A1, 0SG00AJ, 0SG00J0, 0SG00J1, 0SG00JJ, 0SG00K0, 0SG00K1, 0SG00KJ, 0SG00Z0, 0SG00Z1, 0SG00ZJ, 0SG0370, 0SG0371, 0SG037J, 0SG03A0, 0SG03A1, 0SG03AJ, 0SG03J0, 0SG03J1, 0SG03JJ, 0SG03K0, 0SG03K1, 0SG03KJ, 0SG03Z0, 0SG03Z1, 0SG03ZJ, 0SG0470, 0SG0471, 0SG047J, 0SG04A0, 0SG04A1, 0SG04AJ, 0SG04J0, 0SG04J1, 0SG04JJ, 0SG04K0, 0SG04K1, 0SG04KJ, 0SG04Z0, 0SG04Z1, 0SG04ZJ, 0SG1070, 0SG1071, 0SG107J, 0SG10A0, 0SG10A1, 0SG10AJ, 0SG10J0, 0SG10J1, 0SG10JJ, 0SG10K0, 0SG10K1, 0SG10KJ, 0SG10Z0, 0SG10Z1, 0SG10ZJ, 0SG1370, 0SG1371, 0SG137J, 0SG13A0, 0SG13A1, 0SG13AJ, 0SG13J0, 0SG13J1, 0SG13JJ, 0SG13K0, 0SG13K1, 0SG13KJ, 0SG13Z0, 0SG13Z1, 0SG13ZJ, 0SG1470, 0SG1471, 0SG147J, 0SG14A0, 0SG14A1, 0SG14AJ, 0SG14J0, 0SG14J1, 0SG14JJ, 0SG14K0, 0SG14K1, 0SG14KJ, 0SG14Z0, 0SG14Z1, 0SG14ZJ, 0SG3070, 0SG3071, 0SG307J, 0SG30A0, 0SG30A1, 0SG30AJ, 0SG30J0, 0SG30J1, 0SG30JJ, 0SG30K0, 0SG30K1, 0SG30KJ, 0SG30Z0, 0SG30Z1, 0SG30ZJ, 0SG3370, 0SG3371, 0SG337J, 0SG33A0, 0SG33A1, 0SG33AJ, 0SG33J0, 0SG33J1, 0SG33JJ, 0SG33K0, 0SG33K1, 0SG33KJ, 0SG33Z0, 0SG33Z1, 0SG33ZJ, 0SG3470, 0SG3471, 0SG347J, 0SG34A0, 0SG34A1, 0SG34AJ, 0SG34J0, 0SG34J1, 0SG34JJ, 0SG34K0, 0SG34K1, 0SG34KJ, 0SG34Z0, 0SG34Z1, 0SG34ZJ, XRGA092, XRGA0F3, XRGA0R7, XRGA3R7, XRGA4R7, XRGB092, XRGB0F3, XRGB0R7, XRGB3R7, XRGB4R7, XRGC092, XRGC0F3, XRGC0R7, XRGC3R7, XRGC4R7, XRGD092, XRGD0F3, XRGD0R7, XRGD3R7, XRGD4R7

### CPT codes used to define spinal fusion

22612, 22630, 22558, 22586, 22633

### DRG codes used to define spinal fusion

456, 457, 458, 459, 460

ICD-10 DX codes to define other related spine diagnosis

Spondylosis

M47.15, M47.16, M47.25, M47.26, M47.27, M47.815, M47.816, M47.817, M47.895, M47.896, M47.897, M51.15, M51.16, M51.35, M51.36, M51.37

Spondylolysis

M43.05, M43.06, M43.07

Spondylolisthesis

M43.15, M43.16, M43.17, M99.23, M99.63

Radiculopathy

M47.25, M47.26, M47.27, M51.15, M51.16, M51.17, M54.15, M54.16, M54.17, M54.3X, M54.4X

Stenosis

M99.23, M99.33, M99.43, M99.53, M99.63, M99.73, M48.06X, M48.07

### CPT codes that define an index event for the comparison group

#### Physical Therapy Visit Codes

RBCS RT Category (exclude speech therapy)

97001, 97002, 97003, 97004, 97010, 97012, 97014, 97016, 97018, 97022, 97024, 97026, 97028, 97032, 97033, 97034, 97035, 97036, 97039, 97110, 97112, 97113, 97116, 97124, 97139, 97140, 97150, 97161, 97162, 97163, 97164, 97165, 97166, 97167, 97168, 97530, 97532, 97533, 97535, 97537, 97542, 97545, 97546, 97750, 97755, 97760, 97761, 97762, 97763, 97799, 97799, G0129, G0129, G0157, G0501

ICD-10 DX codes that define concurrent MSK conditions

M45.A2, M45.A3, M45.A5, M45.A6, M48.061, M48.062, M50.020, M50.021, M50.022, M50.023, M50.123, M50.120, M50.122, M50.121, M50.223, M50.222, M50.220, M50.221, M50.322, M50.323, M50.321, M50.320, M50.822, M50.823, M50.821, M50.820, M50.923, M50.922, M50.921, M50.920, M51.A2, M51.A0, M51.A1, M54.50, M54.59, M54.51, G54.2, M40.03, M40.05, M40.12, M40.13, M40.15, M40.202, M40.203, M40.205, M40.292, M40.293, M40.295, M40.35, M40.36, M40.45, M40.46, M40.55, M40.56, M41.02, M41.03, M41.05, M41.06, M41.112, M41.113, M41.115, M41.116, M41.122, M41.123, M41.125, M41.126, M41.22, M41.23, M41.25, M41.26, M41.35, M41.42, M41.43, M41.45, M41.46, M41.52, M41.53, M41.55, M41.56, M41.82, M41.83, M41.85, M41.86, M42.02, M42.03, M42.05, M42.06, M42.12, M42.13, M42.15, M42.16, M43.02, M43.03, M43.05, M43.06, M43.12, M43.13, M43.15, M43.16, M43.5X2, M43.5X3, M43.5X5, M43.5X6, M43.8X2, M43.8X3, M43.8X5, M43.8X6, M45.2, M45.3, M45.5, M45.6, M46.02, M46.03, M46.05, M46.06, M46.42, M46.43, M46.45, M46.46, M46.82, M46.83, M46.85, M46.86, M46.92, M46.93, M46.95, M46.96, M47.012, M47.013, M47.015, M47.016, M47.12, M47.13, M47.15, M47.16, M47.22, M47.23, M47.25, M47.26, M47.812, M47.813, M47.815, M47.816, M47.892, M47.893, M47.895, M47.896, M48.02, M48.03, M48.05, M48.06, M48.12, M48.13, M48.15, M48.16, M48.22, M48.23, M48.25, M48.26, M48.42XA, M48.42XD, M48.42XG, M48.42XS, M48.43XA, M48.43XD, M48.43XG, M48.43XS, M48.45XA, M48.45XD, M48.45XG, M48.45XS, M48.46XA, M48.46XD, M48.46XG, M48.46XS, M48.52XA, M48.52XD, M48.52XG, M48.52XS, M48.53XA, M48.53XD, M48.53XG, M48.53XS, M48.55XA, M48.55XD, M48.55XG, M48.55XS, M48.56XA, M48.56XD, M48.56XG, M48.56XS, M49.82, M49.83, M49.86, M50.02, M50.03, M50.12, M50.13, M50.22, M50.23, M50.32, M50.33, M50.82, M50.83, M50.92, M50.93, M51.05, M51.06, M51.15, M51.16, M51.25, M51.26, M51.35, M51.36, M51.85, M51.86, M53.2X5, M53.2X6, M53.82, M53.83, M53.85, M53.86, M54.12, M54.13, M54.15, M54.16, M54.17, M54.2, M54.30, M54.31, M54.32, M54.40, M54.41, M54.42, M54.5, M99.01, M99.03, M99.11, M99.13, M99.21, M99.23, M99.31, M99.33, M99.41, M99.43, M99.51, M99.53, M99.61, M99.63, M99.71, M99.73, M99.81, M99.83

### Exclusion Criteria

| **Excluded conditions** | **CCSR diagnosis codes** |
| --- | --- |
| Cancer, neoplasms | NEO001-NEO025, NEO028-NEO71 |
| Certain conditions originating in the perinatal period | Any PNL code |
| Pregnancy, childbirth, and the puerperium | Any PRG code |

Augmented Inverse Propensity Weighting

The method involves 2 basic steps: first, fitting a propensity score model (i.e., the estimated probability of treatment assignment conditional on observed baseline characteristics), and second, fitting 2 models that estimate the outcome under treatment and control conditions. Each outcome is then weighted by the propensity score from the previous step to produce a weighted average of the 2 outcome models. The AIPW estimator is doubly robust in that it will be consistent for the average treatment effect whenever either (1) the propensity score model is correctly specified or (2) the outcome regression is correctly specified.

Table S1. Augmented Inverse Propensity Weighting

|  | **Estimate** | **SE** | **95% CL lower limit** | **95% CL upper limit** | **N** |
| --- | --- | --- | --- | --- | --- |
| **Risk of exposure** | 0.006 | 0.001 | 0.004 | 0.007 | 3424 |
| **Risk of control** | 0.009 | 0.000 | 0.009 | 0.009 | 220674 |
| **Risk Difference** | -0.003 | 0.001 | -0.005 | -0.002 | 224098 |
| **Risk Ratio** | 0.644 | 0.119 | 0.510 | 0.814 | 224098 |
| **Odds Ratio** | 0.642 | 0.120 | 0.508 | 0.813 | 224098 |

##

Table S2. Multivariable regression model using matched sample

|  |  | **Odds ratio (95% CL, p-value)** | **Adjusted Odds ratio (95% CL, p-value)** |
| --- | --- | --- | --- |
| **Characteristics** |  |  |  |
| **Study group** | Non-participant | - | - |
|  | Digital MSK participant | 0.44 (0.27-0.71, p=0.001) | 0.43 (0.26-0.69, p=0.001) |
| **Age group** | 40-49 | - | - |
|  | 50-64 | 1.96 (1.16-3.52, p=0.017) | 1.90 (1.10-3.46, p=0.027) |
| **Gender** | Male | - | - |
|  | Female | 1.09 (0.69-1.71, p=0.720) | 0.89 (0.55-1.42, p=0.611) |
| **Census region** | New England | - | - |
|  | Middle Atlantic | 0.00 (0.00-648.09, p=0.979) | 0.00 (0.00-26.79, p=0.978) |
|  | East North Central | 0.38 (0.15-1.17, p=0.062) | 0.46 (0.17-1.44, p=0.141) |
|  | West North Central | 0.35 (0.09-1.34, p=0.122) | 0.51 (0.12-2.01, p=0.330) |
|  | South Atlantic | 0.41 (0.16-1.27, p=0.088) | 0.42 (0.15-1.33, p=0.106) |
|  | East South Central | 0.20 (0.03-0.92, p=0.053) | 0.21 (0.03-1.03, p=0.070) |
|  | West South Central | 0.66 (0.27-1.98, p=0.404) | 0.76 (0.29-2.36, p=0.595) |
|  | Mountain | 0.21 (0.04-0.87, p=0.035) | 0.27 (0.05-1.15, p=0.081) |
|  | Pacific | 0.30 (0.11-0.97, p=0.030) | 0.41 (0.14-1.37, p=0.120) |
| **Rural/urban** | Rural | - | - |
|  | Urban | 0.96 (0.50-2.07, p=0.911) | 1.03 (0.52-2.30, p=0.943) |
| **No. of injections (12 mo baseline)** | 0 | - | - |
|  | 1 to 5 | 3.96 (2.41-6.39, p<0.001) | 2.58 (1.39-4.74, p=0.002) |
|  | 6+ | 10.29 (4.39-21.31, p<0.001) | 6.44 (2.34-16.49, p<0.001) |
| **No. of imaging (12 mo baseline)** | 0 | - | - |
|  | 1 to 5 | 3.61 (2.17-6.32, p<0.001) | 2.34 (1.32-4.31, p=0.005) |
|  | 6+ | 8.98 (1.40-32.41, p=0.004) | 2.29 (0.31-10.60, p=0.341) |
| **No. of PT visits (12 mo baseline)** | 0 | - | - |
|  | 1 to 5 | 0.13 (0.04-0.32, p<0.001) | 0.19 (0.06-0.46, p=0.001) |
|  | 6+ | 0.65 (0.37-1.08, p=0.109) | 0.79 (0.44-1.36, p=0.405) |
| **No. of Provider visits (12 mo baseline)** | 0 | - | - |
|  | 1 to 5 | 1.78 (1.00-3.39, p=0.062) | 1.20 (0.65-2.36, p=0.586) |
|  | 6+ | 3.13 (1.17-7.70, p=0.016) | 1.00 (0.33-2.83, p=0.996) |
| **Concurrent MSK** | No | - | - |
|  | Yes | 1.52 (0.97-2.39, p=0.069) | 1.04 (0.65-1.69, p=0.862) |
| **Recent MSK service (3 mo baseline)** | No | - | - |
|  | Yes | 1.10 (0.71-1.73, p=0.663) | 0.88 (0.54-1.42, p=0.607) |
| **Weighted Elixhauser Comorbidity Score** | <0 | - | - |
|  | 0 | 0.64 (0.40-1.05, p=0.071) | 0.67 (0.41-1.12, p=0.120) |
|  | 1-4 | 0.61 (0.18-1.58, p=0.366) | 0.65 (0.19-1.72, p=0.432) |
|  | >=5 | 1.05 (0.06-5.04, p=0.964) | 0.85 (0.05-4.42, p=0.875) |
| **Spondylosis diagnosis** | No | - | - |
|  | Yes | 2.41 (1.54-3.78, p<0.001) | 0.87 (0.50-1.50, p=0.615) |
| **Spondylolysis diagnosis** | No | - | - |
|  | Yes | 0.00 (p=0.984) | 0.00 (p=0.995) |
| **Spondylolisthesis diagnosis** | No | - | - |
|  | Yes | 3.90 (1.61-8.03, p=0.001) | 2.31 (0.91-5.08, p=0.054) |
| **Radiculopathy diagnosis** | No | - | - |
|  | Yes | 2.36 (1.51-3.71, p<0.001) | 1.10 (0.63-1.92, p=0.735) |
| **Stenosis diagnosis** | No | - | - |
|  | Yes | 7.98 (0.43-41.78, p=0.048) | 5.63 (0.29-33.51, p=0.115) |

Table S3. Post-period outcomes, after matching

|  | **Non-participants** | **Digital MSK participants** | **P-value** |
| --- | --- | --- | --- |
| **Post-period outcomes** | (N=3424) | (N=3424) |  |
| **Surgeon visit (12 mo post-period)** |  |  |  |
| 0 | 3030 (88.5%) | 3208 (93.7%) | <0.001 |
| 1 to 5 | 384 (11.2%) | 214 (6.3%) |  |
| 6+ | 10 (0.3%) | 2 (0.1%) |  |
| **Injection (12 mo post-period)** |  |  |  |
| 0 | 2911 (85.0%) | 2995 (87.5%) | 0.0129 |
| 1 to 5 | 452 (13.2%) | 377 (11.0%) |  |
| 6+ | 61 (1.8%) | 52 (1.5%) |  |
| **Imaging (12 mo post-period)** |  |  |  |
| 0 | 2390 (69.8%) | 2741 (80.1%) | <0.001 |
| 1 to 5 | 986 (28.8%) | 665 (19.4%) |  |
| 6+ | 48 (1.4%) | 18 (0.5%) |  |
| **Physical Therapy (12 mo post-period)** |  |  |  |
| 0 | 0 (0%) | 2513 (73.4%) | <0.001 |
| 1 to 5 | 2346 (68.5%) | 449 (13.1%) |  |
| 6+ | 1078 (31.5%) | 462 (13.5%) |  |
| **Provider visit (12 mo post-period)** |  |  |  |
| 0 | 1587 (46.3%) | 2238 (65.4%) | <0.001 |
| 1 to 5 | 1620 (47.3%) | 1041 (30.4%) |  |
| 6+ | 217 (6.3%) | 145 (4.2%) |  |

Figure S1. Love plot to assess covariate balance between groups


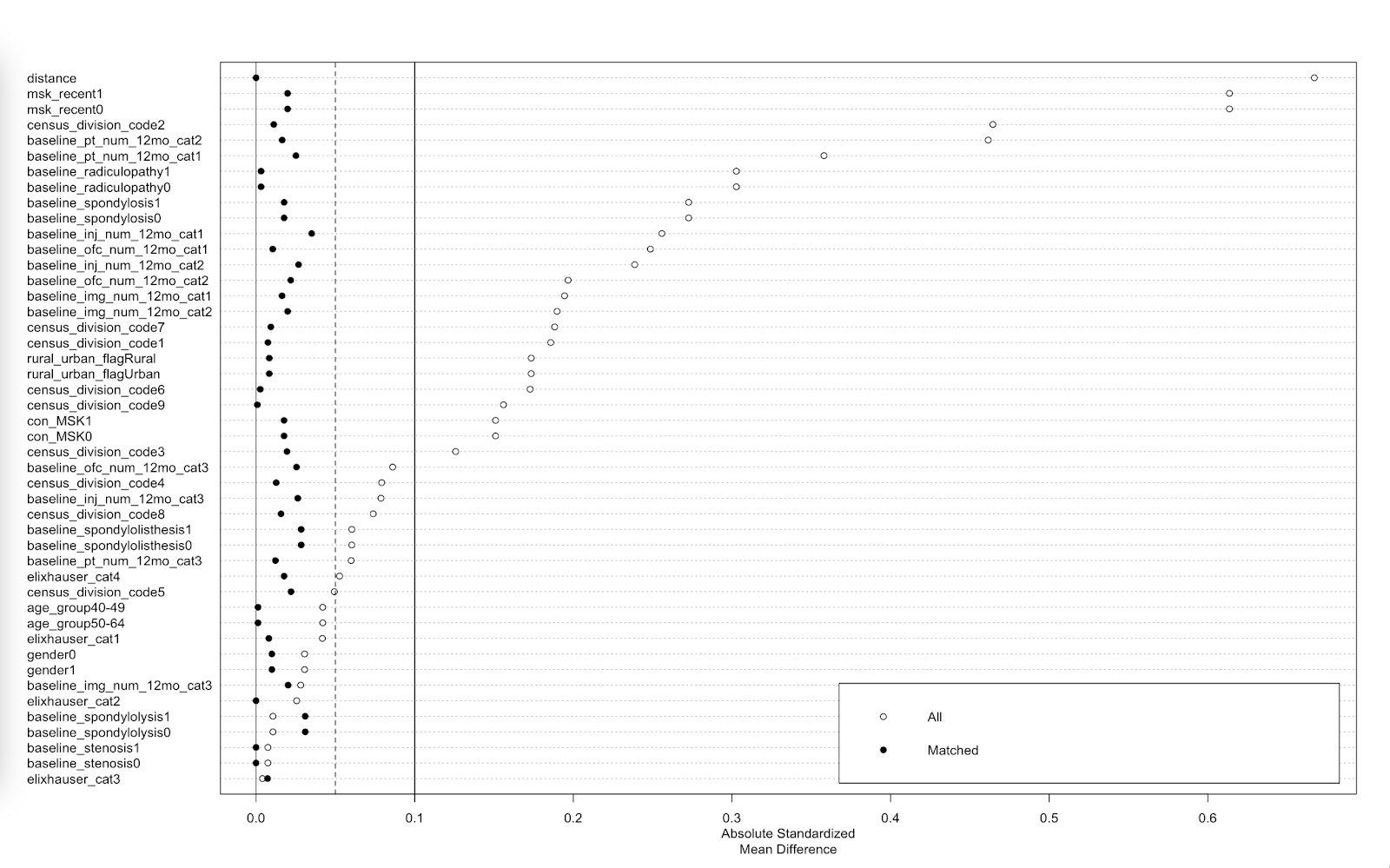

Supplement: Supplementary file 1 — Supplementary Material 1 [file 12891_2024_7573_MOESM1_ESM.docx]
